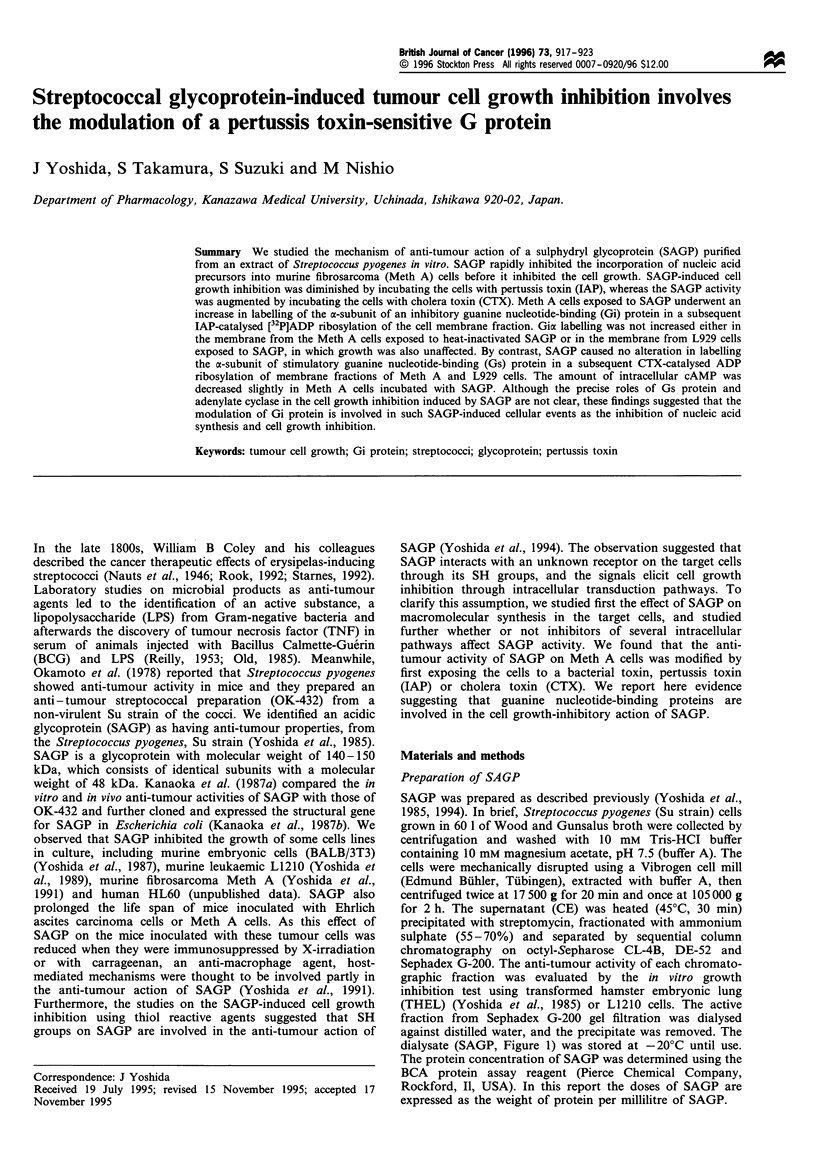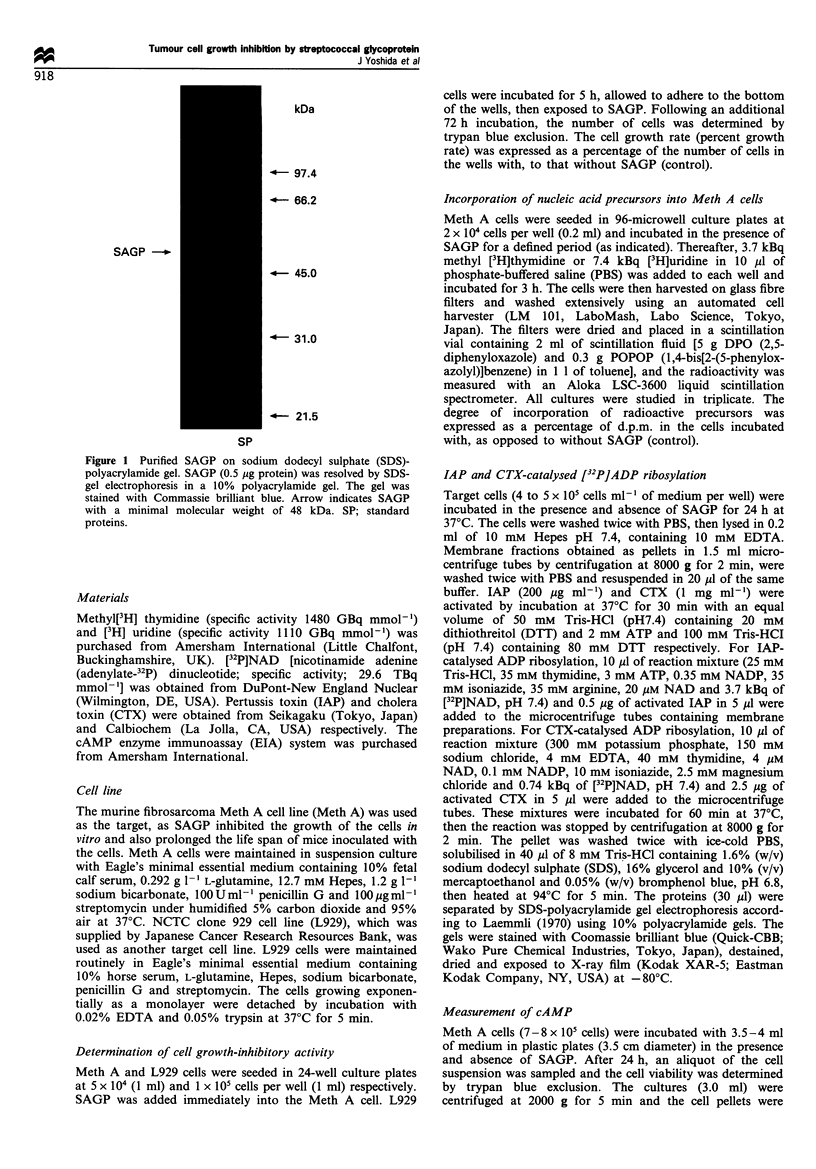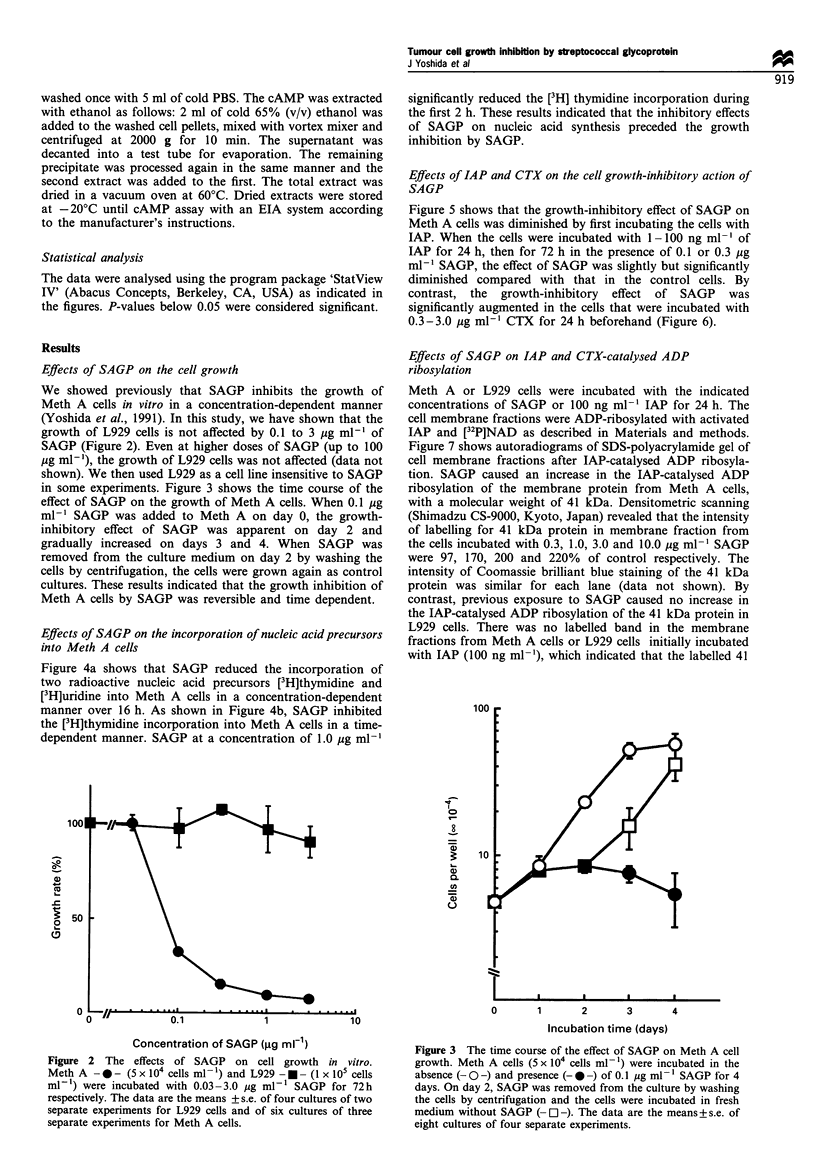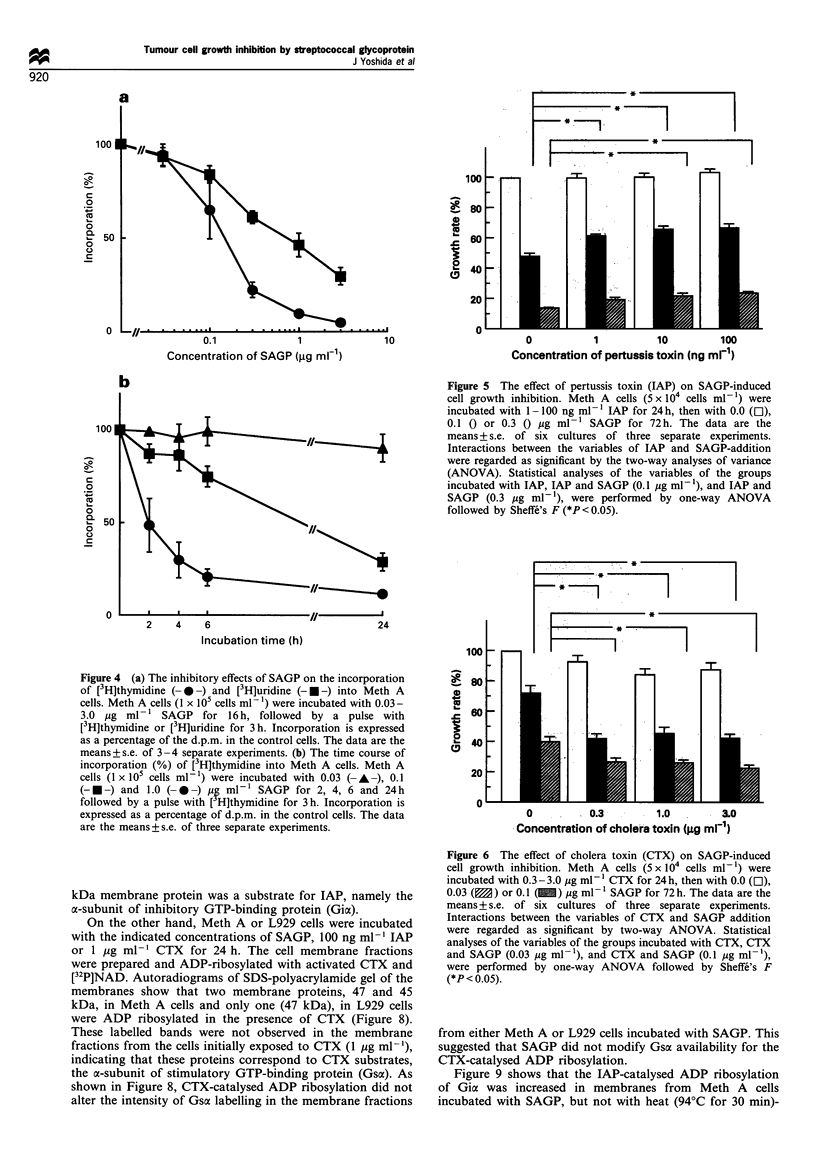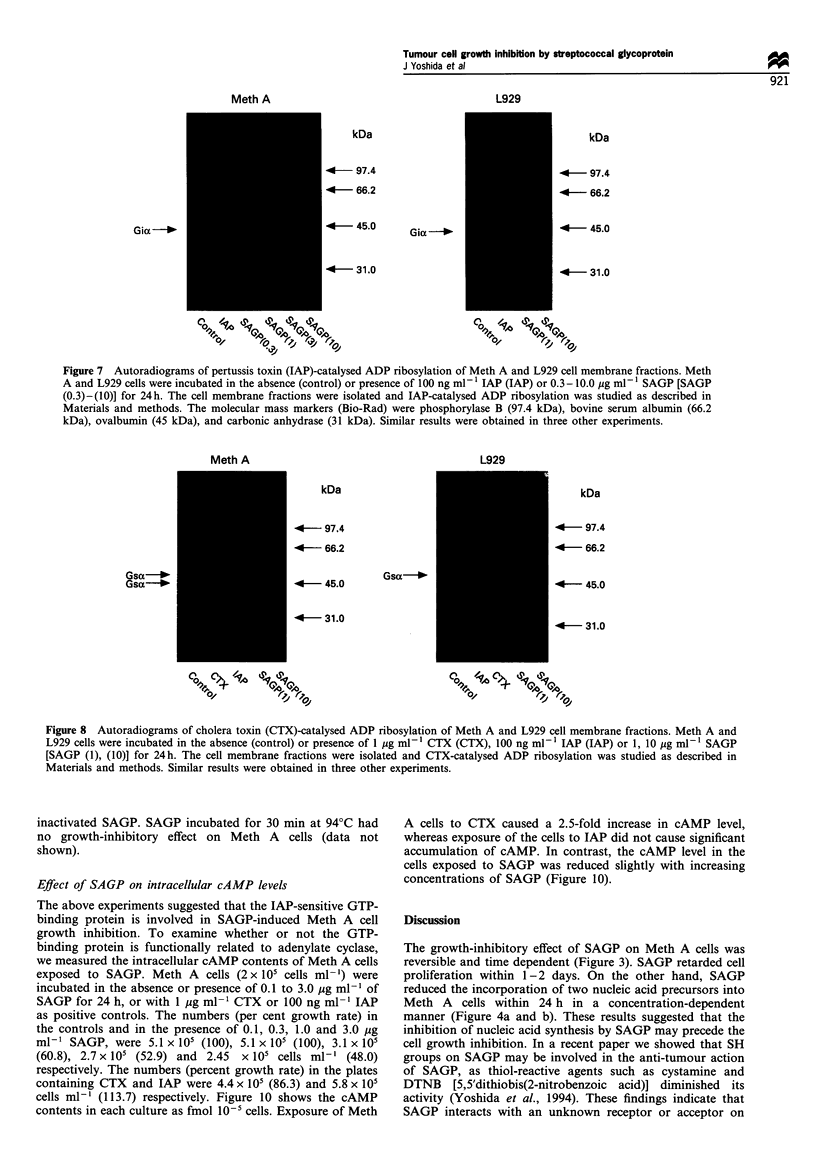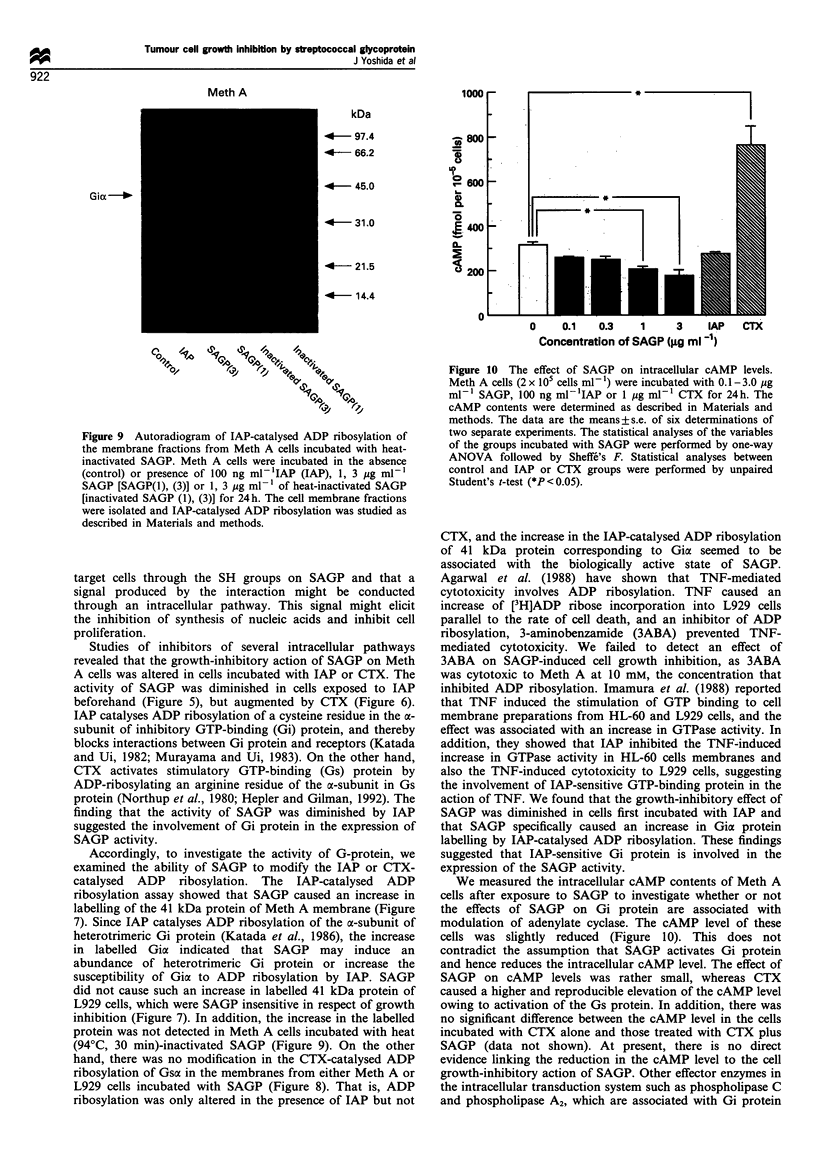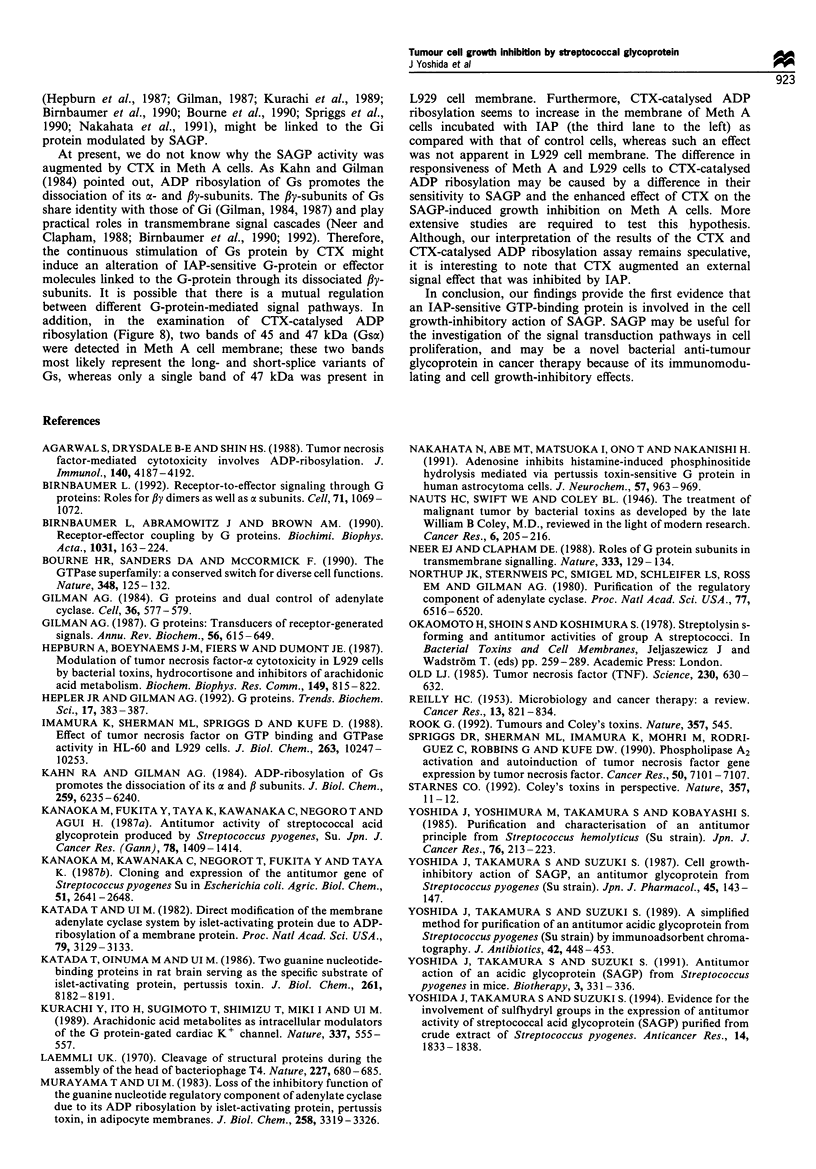# Streptococcal glycoprotein-induced tumour cell growth inhibition involves the modulation of a pertussis toxin-sensitive G protein.

**DOI:** 10.1038/bjc.1996.182

**Published:** 1996-04

**Authors:** J. Yoshida, S. Takamura, S. Suzuki, M. Nishio

**Affiliations:** Department of Pharmacology, Kanazawa Medical University, Ishikawa, Japan.

## Abstract

**Images:**


British Journal of Cancer (1996) 73, 917-923
© 1996 Stockton Press All rights reserved 0007-0920/96 $12.00

### Streptococcal glycoprotein-induced tumour cell growth inhibition involves the modulation of a pertussis toxin-sensitive G protein

J Yoshida, S Takamura, S Suzuki and M Nishio

*Department of Pharmacology, Kanazawa Medical University, Uchinada, Ishikawa 920-02, Japan.*

**Summary** We studied the mechanism of anti-tumour action of a sulphydryl glycoprotein (SAGP) purified from an extract of *Streptococcus pyogenes in vitro*. SAGP rapidly inhibited the incorporation of nucleic acid precursors into murine fibrosarcoma (Meth A) cells before it inhibited the cell growth. SAGP-induced cell growth inhibition was diminished by incubating the cells with pertussis toxin (IAP), whereas the SAGP activity was augmented by incubating the cells with cholera toxin (CTX). Meth A cells exposed to SAGP underwent an increase in labelling of the α-subunit of an inhibitory guanine nucleotide-binding (Gi) protein in a subsequent IAP-catalysed [$^{32}$P]ADP ribosylation of the cell membrane fraction. Giα labelling was not increased either in the membrane from the Meth A cells exposed to heat-inactivated SAGP or in the membrane from L929 cells exposed to SAGP, in which growth was also unaffected. By contrast, SAGP caused no alteration in labelling the α-subunit of stimulatory guanine nucleotide-binding (Gs) protein in a subsequent CTX-catalysed ADP ribosylation of membrane fractions of Meth A and L929 cells. The amount of intracellular cAMP was decreased slightly in Meth A cells incubated with SAGP. Although the precise roles of Gs protein and adenylate cyclase in the cell growth inhibition induced by SAGP are not clear, these findings suggested that the modulation of Gi protein is involved in such SAGP-induced cellular events as the inhibition of nucleic acid synthesis and cell growth inhibition.

**Keywords:** tumour cell growth; Gi protein; streptococci; glycoprotein; pertussis toxin

In the late 1800s, William B Coley and his colleagues described the cancer therapeutic effects of erysipelas-inducing streptococci (Nauts *et al.*, 1946; Rook, 1992; Starnes, 1992). Laboratory studies on microbial products as anti-tumour agents led to the identification of an active substance, a lipopolysaccharide (LPS) from Gram-negative bacteria and afterwards the discovery of tumour necrosis factor (TNF) in serum of animals injected with Bacillus Calmette-Guérin (BCG) and LPS (Reilly, 1953; Old, 1985). Meanwhile, Okamoto *et al.* (1978) reported that *Streptococcus pyogenes* showed anti-tumour activity in mice and they prepared an anti–tumour streptococcal preparation (OK-432) from a non-virulent Su strain of the cocci. We identified an acidic glycoprotein (SAGP) as having anti-tumour properties, from the *Streptococcus pyogenes*, Su strain (Yoshida *et al.*, 1985). SAGP is a glycoprotein with molecular weight of 140–150 kDa, which consists of identical subunits with a molecular weight of 48 kDa. Kanaoka *et al.* (1987*a*) compared the *in vitro* and *in vivo* anti-tumour activities of SAGP with those of OK-432 and further cloned and expressed the structural gene for SAGP in *Escherichia coli* (Kanaoka *et al.*, 1987*b*). We observed that SAGP inhibited the growth of some cells lines in culture, including murine embryonic cells (BALB/3T3) (Yoshida *et al.*, 1987), murine leukaemic L1210 (Yoshida *et al.*, 1989), murine fibrosarcoma Meth A (Yoshida *et al.*, 1991) and human HL60 (unpublished data). SAGP also prolonged the life span of mice inoculated with Ehrlich ascites carcinoma cells or Meth A cells. As this effect of SAGP on the mice inoculated with these tumour cells was reduced when they were immunosuppressed by X-irradiation or with carrageenan, an anti-macrophage agent, host-mediated mechanisms were thought to be involved partly in the anti-tumour action of SAGP (Yoshida *et al.*, 1991). Furthermore, the studies on the SAGP-induced cell growth inhibition using thiol reactive agents suggested that SH groups on SAGP are involved in the anti-tumour action of SAGP (Yoshida *et al.*, 1994). The observation suggested that SAGP interacts with an unknown receptor on the target cells through its SH groups, and the signals elicit cell growth inhibition through intracellular transduction pathways. To clarify this assumption, we studied first the effect of SAGP on macromolecular synthesis in the target cells, and studied further whether or not inhibitors of several intracellular pathways affect SAGP activity. We found that the anti-tumour activity of SAGP on Meth A cells was modified by first exposing the cells to a bacterial toxin, pertussis toxin (IAP) or cholera toxin (CTX). We report here evidence suggesting that guanine nucleotide-binding proteins are involved in the cell growth-inhibitory action of SAGP.

Correspondence: J Yoshida
Received 19 July 1995; revised 15 November 1995; accepted 17 November 1995

### Materials and methods

### *Preparation of SAGP*

SAGP was prepared as described previously (Yoshida *et al.*, 1985, 1994). In brief, *Streptococcus pyogenes* (Su strain) cells grown in 60 l of Wood and Gunsalus broth were collected by centrifugation and washed with 10 mM Tris-HCl buffer containing 10 mM magnesium acetate, pH 7.5 (buffer A). The cells were mechanically disrupted using a Vibrogen cell mill (Edmund Bühler, Tübingen), extracted with buffer A, then centrifuged twice at 17 500 g for 20 min and once at 105 000 g for 2 h. The supernatant (CE) was heated (45°C, 30 min) precipitated with streptomycin, fractionated with ammonium sulphate (55–70%) and separated by sequential column chromatography on octyl-*S*epharose CL-4B, DE-52 and Sephadex G-200. The anti-tumour activity of each chromatographic fraction was evaluated by the *in vitro* growth inhibition test using transformed hamster embryonic lung (THEL) (Yoshida *et al.*, 1985) or L1210 cells. The active fraction from Sephadex G-200 gel filtration was dialysed against distilled water, and the precipitate was removed. The dialysate (SAGP, Figure 1) was stored at −20°C until use. The protein concentration of SAGP was determined using the BCA protein assay reagent (Pierce Chemical Company, Rockford, Il, USA). In this report the doses of SAGP are expressed as the weight of protein per millilitre of SAGP.

**Figure 1** Purified SAGP on sodium dodecyl sulphate (SDS)-polyacrylamide gel. SAGP (0.5 $\mu$g protein) was resolved by SDS-gel electrophoresis in a 10% polyacrylamide gel. The gel was stained with Commassie brilliant blue. Arrow indicates SAGP with a minimal molecular weight of 48 kDa. SP; standard proteins.

*Materials*

Methyl[$^3$H] thymidine (specific activity 1480 GBq mmol$^{-1}$) and [$^3$H] uridine (specific activity 1110 GBq mmol$^{-1}$) was purchased from Amersham International (Little Chalfont, Buckinghamshire, UK). [$^{32}$P]NAD [nicotinamide adenine (adenylate-$^{32}$P) dinucleotide; specific activity; 29.6 TBq mmol$^{-1}$] was obtained from DuPont-New England Nuclear (Wilmington, DE, USA). Pertussis toxin (IAP) and cholera toxin (CTX) were obtained from Seikagaku (Tokyo, Japan) and Calbiochem (La Jolla, CA, USA) respectively. The cAMP enzyme immunoassay (EIA) system was purchased from Amersham International.

*Cell line*

The murine fibrosarcoma Meth A cell line (Meth A) was used as the target, as SAGP inhibited the growth of the cells *in vitro* and also prolonged the life span of mice inoculated with the cells. Meth A cells were maintained in suspension culture with Eagle's minimal essential medium containing 10% fetal calf serum, 0.292 g l$^{-1}$ L-glutamine, 12.7 mM Hepes, 1.2 g l$^{-1}$ sodium bicarbonate, 100 U ml$^{-1}$ penicillin G and 100 $\mu$g ml$^{-1}$ streptomycin under humidified 5% carbon dioxide and 95% air at 37°C. NCTC clone 929 cell line (L929), which was supplied by Japanese Cancer Research Resources Bank, was used as another target cell line. L929 cells were maintained routinely in Eagle's minimal essential medium containing 10% horse serum, L-glutamine, Hepes, sodium bicarbonate, penicillin G and streptomycin. The cells growing exponentially as a monolayer were detached by incubation with 0.02% EDTA and 0.05% trypsin at 37°C for 5 min.

*Determination of cell growth-inhibitory activity*

Meth A and L929 cells were seeded in 24-well culture plates at $5 \times 10^4$ (1 ml) and $1 \times 10^5$ cells per well (1 ml) respectively. SAGP was added immediately into the Meth A cell. L929 cells were incubated for 5 h, allowed to adhere to the bottom of the wells, then exposed to SAGP. Following an additional 72 h incubation, the number of cells was determined by trypan blue exclusion. The cell growth rate (percent growth rate) was expressed as a percentage of the number of cells in the wells with, to that without SAGP (control).

*Incorporation of nucleic acid precursors into Meth A cells*

Meth A cells were seeded in 96-microwell culture plates at $2 \times 10^4$ cells per well (0.2 ml) and incubated in the presence of SAGP for a defined period (as indicated). Thereafter, 3.7 kBq methyl [$^3$H]thymidine or 7.4 kBq [$^3$H]uridine in 10 $\mu$l of phosphate-buffered saline (PBS) was added to each well and incubated for 3 h. The cells were then harvested on glass fibre filters and washed extensively using an automated cell harvester (LM 101, LaboMash, Labo Science, Tokyo, Japan). The filters were dried and placed in a scintillation vial containing 2 ml of scintillation fluid [5 g DPO (2,5-diphenyloxazole) and 0.3 g POPOP (1,4-bis[2-(5-phenyloxazolyl)]benzene) in 1 l of toluene], and the radioactivity was measured with an Aloka LSC-3600 liquid scintillation spectrometer. All cultures were studied in triplicate. The degree of incorporation of radioactive precursors was expressed as a percentage of d.p.m. in the cells incubated with, as opposed to without SAGP (control).

*IAP and CTX-catalysed [$^{32}$P]ADP ribosylation*

Target cells (4 to $5 \times 10^5$ cells ml$^{-1}$ of medium per well) were incubated in the presence and absence of SAGP for 24 h at 37°C. The cells were washed twice with PBS, then lysed in 0.2 ml of 10 mM Hepes pH 7.4, containing 10 mM EDTA. Membrane fractions obtained as pellets in 1.5 ml microcentrifuge tubes by centrifugation at 8000 **g** for 2 min, were washed twice with PBS and resuspended in 20 $\mu$l of the same buffer. IAP (200 $\mu$g ml$^{-1}$) and CTX (1 mg ml$^{-1}$) were activated by incubation at 37°C for 30 min with an equal volume of 50 mM Tris-HCl (pH7.4) containing 20 mM dithiothreitol (DTT) and 2 mM ATP and 100 mM Tris-HCl (pH 7.4) containing 80 mM DTT respectively. For IAP-catalysed ADP ribosylation, 10 $\mu$l of reaction mixture (25 mM Tris-HCl, 35 mM thymidine, 3 mM ATP, 0.35 mM NADP, 35 mM isoniazide, 35 mM arginine, 20 $\mu$M NAD and 3.7 kBq of [$^{32}$P]NAD, pH 7.4) and 0.5 $\mu$g of activated IAP in 5 $\mu$l were added to the microcentrifuge tubes containing membrane preparations. For CTX-catalysed ADP ribosylation, 10 $\mu$l of reaction mixture (300 mM potassium phosphate, 150 mM sodium chloride, 4 mM EDTA, 40 mM thymidine, 4 $\mu$M NAD, 0.1 mM NADP, 10 mM isoniazide, 2.5 mM magnesium chloride and 0.74 kBq of [$^{32}$P]NAD, pH 7.4) and 2.5 $\mu$g of activated CTX in 5 $\mu$l were added to the microcentrifuge tubes. These mixtures were incubated for 60 min at 37°C, then the reaction was stopped by centrifugation at 8000 **g** for 2 min. The pellet was washed twice with ice-cold PBS, solubilised in 40 $\mu$l of 8 mM Tris-HCl containing 1.6% (w/v) sodium dodecyl sulphate (SDS), 16% glycerol and 10% (v/v) mercaptoethanol and 0.05% (w/v) bromphenol blue, pH 6.8, then heated at 94°C for 5 min. The proteins (30 $\mu$l) were separated by SDS-polyacrylamide gel electrophoresis according to Laemmli (1970) using 10% polyacrylamide gels. The gels were stained with Coomassie brilliant blue (Quick-CBB; Wako Pure Chemical Industries, Tokyo, Japan), destained, dried and exposed to X-ray film (Kodak XAR-5; Eastman Kodak Company, NY, USA) at −80°C.

*Measurement of cAMP*

Meth A cells ($7-8 \times 10^5$ cells) were incubated with 3.5–4 ml of medium in plastic plates (3.5 cm diameter) in the presence and absence of SAGP. After 24 h, an aliquot of the cell suspension was sampled and the cell viability was determined by trypan blue exclusion. The cultures (3.0 ml) were centrifuged at 2000 **g** for 5 min and the cell pellets were washed once with 5 ml of cold PBS. The cAMP was extracted with ethanol as follows: 2 ml of cold 65% (v/v) ethanol was added to the washed cell pellets, mixed with vortex mixer and centrifuged at 2000 g for 10 min. The supernatant was decanted into a test tube for evaporation. The remaining precipitate was processed again in the same manner and the second extract was added to the first. The total extract was dried in a vacuum oven at 60°C. Dried extracts were stored at −20°C until cAMP assay with an EIA system according to the manufacturer's instructions.

*Statistical analysis*

The data were analysed using the program package 'StatView IV' (Abacus Concepts, Berkeley, CA, USA) as indicated in the figures. *P*-values below 0.05 were considered significant.

### Results

*Effects of SAGP on the cell growth*

We showed previously that SAGP inhibits the growth of Meth A cells *in vitro* in a concentration-dependent manner (Yoshida *et al.*, 1991). In this study, we have shown that the growth of L929 cells is not affected by 0.1 to 3 $\mu$g ml$^{-1}$ of SAGP (Figure 2). Even at higher doses of SAGP (up to 100 $\mu$g ml$^{-1}$), the growth of L929 cells was not affected (data not shown). We then used L929 as a cell line insensitive to SAGP in some experiments. Figure 3 shows the time course of the effect of SAGP on the growth of Meth A cells. When 0.1 $\mu$g ml$^{-1}$ SAGP was added to Meth A on day 0, the growth-inhibitory effect of SAGP was apparent on day 2 and gradually increased on days 3 and 4. When SAGP was removed from the culture medium on day 2 by washing the cells by centrifugation, the cells were grown again as control cultures. These results indicated that the growth inhibition of Meth A cells by SAGP was reversible and time dependent.

*Effects of SAGP on the incorporation of nucleic acid precursors into Meth A cells*

Figure 4a shows that SAGP reduced the incorporation of two radioactive nucleic acid precursors [$^3$H]thymidine and [$^3$H]uridine into Meth A cells in a concentration-dependent manner over 16 h. As shown in Figure 4b, SAGP inhibited the [$^3$H]thymidine incorporation into Meth A cells in a time-dependent manner. SAGP at a concentration of 1.0 $\mu$g ml$^{-1}$ significantly reduced the [$^3$H] thymidine incorporation during the first 2 h. These results indicated that the inhibitory effects of SAGP on nucleic acid synthesis preceded the growth inhibition by SAGP.

*Effects of IAP and CTX on the cell growth-inhibitory action of SAGP*

Figure 5 shows that the growth-inhibitory effect of SAGP on Meth A cells was diminished by first incubating the cells with IAP. When the cells were incubated with 1−100 ng ml$^{-1}$ of IAP for 24 h, then for 72 h in the presence of 0.1 or 0.3 $\mu$g ml$^{-1}$ SAGP, the effect of SAGP was slightly but significantly diminished compared with that in the control cells. By contrast, the growth-inhibitory effect of SAGP was significantly augmented in the cells that were incubated with 0.3−3.0 $\mu$g ml$^{-1}$ CTX for 24 h beforehand (Figure 6).

*Effects of SAGP on IAP and CTX-catalysed ADP ribosylation*

Meth A or L929 cells were incubated with the indicated concentrations of SAGP or 100 ng ml$^{-1}$ IAP for 24 h. The cell membrane fractions were ADP-ribosylated with activated IAP and [$^{32}$P]NAD as described in Materials and methods. Figure 7 shows autoradiograms of SDS-polyacrylamide gel of cell membrane fractions after IAP-catalysed ADP ribosylation. SAGP caused an increase in the IAP-catalysed ADP ribosylation of the membrane protein from Meth A cells, with a molecular weight of 41 kDa. Densitometric scanning (Shimadzu CS-9000, Kyoto, Japan) revealed that the intensity of labelling for 41 kDa protein in membrane fraction from the cells incubated with 0.3, 1.0, 3.0 and 10.0 $\mu$g ml$^{-1}$ SAGP were 97, 170, 200 and 220% of control respectively. The intensity of Coomassie brilliant blue staining of the 41 kDa protein was similar for each lane (data not shown). By contrast, previous exposure to SAGP caused no increase in the IAP-catalysed ADP ribosylation of the 41 kDa protein in L929 cells. There was no labelled band in the membrane fractions from Meth A cells or L929 cells initially incubated with IAP (100 ng ml$^{-1}$), which indicated that the labelled 41

**Figure 2** The effects of SAGP on cell growth *in vitro*. Meth A -●- ($5 \times 10^4$ cells ml$^{-1}$) and L929 -■- ($1 \times 10^5$ cells ml$^{-1}$) were incubated with 0.03−3.0 $\mu$g ml$^{-1}$ SAGP for 72 h respectively. The data are the means ±s.e. of four cultures of two separate experiments for L929 cells and of six cultures of three separate experiments for Meth A cells.

**Figure 3** The time course of the effect of SAGP on Meth A cell growth. Meth A cells ($5 \times 10^4$ cells ml$^{-1}$) were incubated in the absence (-○-) and presence (-●-) of 0.1 $\mu$g ml$^{-1}$ SAGP for 4 days. On day 2, SAGP was removed from the culture by washing the cells by centrifugation and the cells were incubated in fresh medium without SAGP (-□-). The data are the means±s.e. of eight cultures of four separate experiments.

**Figure 4** (**a**) The inhibitory effects of SAGP on the incorporation of [$^3$H]thymidine (–●–) and [$^3$H]uridine (–■–) into Meth A cells. Meth A cells ($1 \times 10^5$ cells ml$^{-1}$) were incubated with 0.03–3.0 μg ml$^{-1}$ SAGP for 16 h, followed by a pulse with [$^3$H]thymidine or [$^3$H]uridine for 3 h. Incorporation is expressed as a percentage of the d.p.m. in the control cells. The data are the means ± s.e. of 3–4 separate experiments. (**b**) The time course of incorporation (%) of [$^3$H]thymidine into Meth A cells. Meth A cells ($1 \times 10^5$ cells ml$^{-1}$) were incubated with 0.03 (–▲–), 0.1 (–■–) and 1.0 (–●–) μg ml$^{-1}$ SAGP for 2, 4, 6 and 24 h followed by a pulse with [$^3$H]thymidine for 3 h. Incorporation is expressed as a percentage of d.p.m. in the control cells. The data are the means ± s.e. of three separate experiments.

**Figure 5** The effect of pertussis toxin (IAP) on SAGP-induced cell growth inhibition. Meth A cells ($5 \times 10^4$ cells ml$^{-1}$) were incubated with 1–100 ng ml$^{-1}$ IAP for 24 h, then with 0.0 (□), 0.1 () or 0.3 () μg ml$^{-1}$ SAGP for 72 h. The data are the means ± s.e. of six cultures of three separate experiments. Interactions between the variables of IAP and SAGP-addition were regarded as significant by the two-way analyses of variance (ANOVA). Statistical analyses of the variables of the groups incubated with IAP, IAP and SAGP (0.1 μg ml$^{-1}$), and IAP and SAGP (0.3 μg ml$^{-1}$), were performed by one-way ANOVA followed by Sheffé's $F$ ($^*P < 0.05$).

**Figure 6** The effect of cholera toxin (CTX) on SAGP-induced cell growth inhibition. Meth A cells ($5 \times 10^4$ cells ml$^{-1}$) were incubated with 0.3–3.0 μg ml$^{-1}$ CTX for 24 h, then with 0.0 (□), 0.03 (▨) or 0.1 (■) μg ml$^{-1}$ SAGP for 72 h. The data are the means ± s.e. of six cultures of three separate experiments. Interactions between the variables of CTX and SAGP addition were regarded as significant by two-way ANOVA. Statistical analyses of the variables of the groups incubated with CTX, CTX and SAGP (0.03 μg ml$^{-1}$), and CTX and SAGP (0.1 μg ml$^{-1}$), were performed by one-way ANOVA followed by Sheffé's $F$ ($^*P < 0.05$).

kDa membrane protein was a substrate for IAP, namely the α-subunit of inhibitory GTP-binding protein (Giα).

On the other hand, Meth A or L929 cells were incubated with the indicated concentrations of SAGP, 100 ng ml$^{-1}$ IAP or 1 μg ml$^{-1}$ CTX for 24 h. The cell membrane fractions were prepared and ADP-ribosylated with activated CTX and [$^{32}$P]NAD. Autoradiograms of SDS-polyacrylamide gel of the membranes show that two membrane proteins, 47 and 45 kDa, in Meth A cells and only one (47 kDa), in L929 cells were ADP ribosylated in the presence of CTX (Figure 8). These labelled bands were not observed in the membrane fractions from the cells initially exposed to CTX (1 μg ml$^{-1}$), indicating that these proteins correspond to CTX substrates, the α-subunit of stimulatory GTP-binding protein (Gsα). As shown in Figure 8, CTX-catalysed ADP ribosylation did not alter the intensity of Gsα labelling in the membrane fractions from either Meth A or L929 cells incubated with SAGP. This suggested that SAGP did not modify Gsα availability for the CTX-catalysed ADP ribosylation.

Figure 9 shows that the IAP-catalysed ADP ribosylation of Giα was increased in membranes from Meth A cells incubated with SAGP, but not with heat (94°C for 30 min)-

**Figure 7** Autoradiograms of pertussis toxin (IAP)-catalysed ADP ribosylation of Meth A and L929 cell membrane fractions. Meth A and L929 cells were incubated in the absence (control) or presence of 100 ng $ml^{-1}$ IAP (IAP) or 0.3–10.0 $\mu g$ $ml^{-1}$ SAGP [SAGP (0.3)–(10)] for 24 h. The cell membrane fractions were isolated and IAP-catalysed ADP ribosylation was studied as described in Materials and methods. The molecular mass markers (Bio-Rad) were phosphorylase B (97.4 kDa), bovine serum albumin (66.2 kDa), ovalbumin (45 kDa), and carbonic anhydrase (31 kDa). Similar results were obtained in three other experiments.

**Figure 8** Autoradiograms of cholera toxin (CTX)-catalysed ADP ribosylation of Meth A and L929 cell membrane fractions. Meth A and L929 cells were incubated in the absence (control) or presence of 1 $\mu g$ $ml^{-1}$ CTX (CTX), 100 ng $ml^{-1}$ IAP (IAP) or 1, 10 $\mu g$ $ml^{-1}$ SAGP [SAGP (1), (10)] for 24 h. The cell membrane fractions were isolated and CTX-catalysed ADP ribosylation was studied as described in Materials and methods. Similar results were obtained in three other experiments.

inactivated SAGP. SAGP incubated for 30 min at 94°C had no growth-inhibitory effect on Meth A cells (data not shown).

### *Effect of SAGP on intracellular cAMP levels*

The above experiments suggested that the IAP-sensitive GTP-binding protein is involved in SAGP-induced Meth A cell growth inhibition. To examine whether or not the GTP-binding protein is functionally related to adenylate cyclase, we measured the intracellular cAMP contents of Meth A cells exposed to SAGP. Meth A cells ($2 \times 10^5$ cells $ml^{-1}$) were incubated in the absence or presence of 0.1 to 3.0 $\mu g$ $ml^{-1}$ of SAGP for 24 h, or with 1 $\mu g$ $ml^{-1}$ CTX or 100 ng $ml^{-1}$ IAP as positive controls. The numbers (per cent growth rate) in the controls and in the presence of 0.1, 0.3, 1.0 and 3.0 $\mu g$ $ml^{-1}$ SAGP, were $5.1 \times 10^5$ (100), $5.1 \times 10^5$ (100), $3.1 \times 10^5$ (60.8), $2.7 \times 10^5$ (52.9) and 2.45 $\times 10^5$ cells $ml^{-1}$ (48.0) respectively. The numbers (percent growth rate) in the plates containing CTX and IAP were $4.4 \times 10^5$ (86.3) and $5.8 \times 10^5$ cells $ml^{-1}$ (113.7) respectively. Figure 10 shows the cAMP contents in each culture as fmol $10^{-5}$ cells. Exposure of Meth A cells to CTX caused a 2.5-fold increase in cAMP level, whereas exposure of the cells to IAP did not cause significant accumulation of cAMP. In contrast, the cAMP level in the cells exposed to SAGP was reduced slightly with increasing concentrations of SAGP (Figure 10).

### Discussion

The growth-inhibitory effect of SAGP on Meth A cells was reversible and time dependent (Figure 3). SAGP retarded cell proliferation within 1–2 days. On the other hand, SAGP reduced the incorporation of two nucleic acid precursors into Meth A cells within 24 h in a concentration-dependent manner (Figure 4a and b). These results suggested that the inhibition of nucleic acid synthesis by SAGP may precede the cell growth inhibition. In a recent paper we showed that SH groups on SAGP may be involved in the anti-tumour action of SAGP, as thiol-reactive agents such as cystamine and DTNB [5,5′dithiobis(2-nitrobenzoic acid)] diminished its activity (Yoshida *et al.*, 1994). These findings indicate that SAGP interacts with an unknown receptor or acceptor on

**Figure 9** Autoradiogram of IAP-catalysed ADP ribosylation of the membrane fractions from Meth A cells incubated with heat-inactivated SAGP. Meth A cells were incubated in the absence (control) or presence of 100 ng $ml^{-1}$IAP (IAP), 1, 3 $\mu g\ ml^{-1}$ SAGP [SAGP(1), (3)] or 1, 3 $\mu g\ ml^{-1}$ of heat-inactivated SAGP [inactivated SAGP (1), (3)] for 24 h. The cell membrane fractions were isolated and IAP-catalysed ADP ribosylation was studied as described in Materials and methods.

**Figure 10** The effect of SAGP on intracellular cAMP levels. Meth A cells ($2 \times 10^5$ cells $ml^{-1}$) were incubated with 0.1–3.0 $\mu g\ ml^{-1}$ SAGP, 100 ng $ml^{-1}$IAP or 1 $\mu g\ ml^{-1}$ CTX for 24 h. The cAMP contents were determined as described in Materials and methods. The data are the means$\pm$s.e. of six determinations of two separate experiments. The statistical analyses of the variables of the groups incubated with SAGP were performed by one-way ANOVA followed by Sheffé's *F*. Statistical analyses between control and IAP or CTX groups were performed by unpaired Student's *t*-test ($*P < 0.05$).

target cells through the SH groups on SAGP and that a signal produced by the interaction might be conducted through an intracellular pathway. This signal might elicit the inhibition of synthesis of nucleic acids and inhibit cell proliferation.

Studies of inhibitors of several intracellular pathways revealed that the growth-inhibitory action of SAGP on Meth A cells was altered in cells incubated with IAP or CTX. The activity of SAGP was diminished in cells exposed to IAP beforehand (Figure 5), but augmented by CTX (Figure 6). IAP catalyses ADP ribosylation of a cysteine residue in the α-subunit of inhibitory GTP-binding (Gi) protein, and thereby blocks interactions between Gi protein and receptors (Katada and Ui, 1982; Murayama and Ui, 1983). On the other hand, CTX activates stimulatory GTP-binding (Gs) protein by ADP-ribosylating an arginine residue of the α-subunit in Gs protein (Northup *et al.*, 1980; Hepler and Gilman, 1992). The finding that the activity of SAGP was diminished by IAP suggested the involvement of Gi protein in the expression of SAGP activity.

Accordingly, to investigate the activity of G-protein, we examined the ability of SAGP to modify the IAP or CTX-catalysed ADP ribosylation. The IAP-catalysed ADP ribosylation assay showed that SAGP caused an increase in labelling of the 41 kDa protein of Meth A membrane (Figure 7). Since IAP catalyses ADP ribosylation of the α-subunit of heterotrimeric Gi protein (Katada *et al.*, 1986), the increase in labelled Giα indicated that SAGP may induce an abundance of heterotrimeric Gi protein or increase the susceptibility of Giα to ADP ribosylation by IAP. SAGP did not cause such an increase in labelled 41 kDa protein of L929 cells, which were SAGP insensitive in respect of growth inhibition (Figure 7). In addition, the increase in the labelled protein was not detected in Meth A cells incubated with heat (94°C, 30 min)-inactivated SAGP (Figure 9). On the other hand, there was no modification in the CTX-catalysed ADP ribosylation of Gsα in the membranes from either Meth A or L929 cells incubated with SAGP (Figure 8). That is, ADP ribosylation was only altered in the presence of IAP but not CTX, and the increase in the IAP-catalysed ADP ribosylation of 41 kDa protein corresponding to Giα seemed to be associated with the biologically active state of SAGP. Agarwal *et al.* (1988) have shown that TNF-mediated cytotoxicity involves ADP ribosylation. TNF caused an increase of [$^3$H]ADP ribose incorporation into L929 cells parallel to the rate of cell death, and an inhibitor of ADP ribosylation, 3-aminobenzamide (3ABA) prevented TNF-mediated cytotoxicity. We failed to detect an effect of 3ABA on SAGP-induced cell growth inhibition, as 3ABA was cytotoxic to Meth A at 10 mM, the concentration that inhibited ADP ribosylation. Imamura *et al.* (1988) reported that TNF induced the stimulation of GTP binding to cell membrane preparations from HL-60 and L929 cells, and the effect was associated with an increase in GTPase activity. In addition, they showed that IAP inhibited the TNF-induced increase in GTPase activity in HL-60 cells membranes and also the TNF-induced cytotoxicity to L929 cells, suggesting the involvement of IAP-sensitive GTP-binding protein in the action of TNF. We found that the growth-inhibitory effect of SAGP was diminished in cells first incubated with IAP and that SAGP specifically caused an increase in Giα protein labelling by IAP-catalysed ADP ribosylation. These findings suggested that IAP-sensitive Gi protein is involved in the expression of the SAGP activity.

We measured the intracellular cAMP contents of Meth A cells after exposure to SAGP to investigate whether or not the effects of SAGP on Gi protein are associated with modulation of adenylate cyclase. The cAMP level of these cells was slightly reduced (Figure 10). This does not contradict the assumption that SAGP activates Gi protein and hence reduces the intracellular cAMP level. The effect of SAGP on cAMP levels was rather small, whereas CTX caused a higher and reproducible elevation of the cAMP level owing to activation of the Gs protein. In addition, there was no significant difference between the cAMP level in the cells incubated with CTX alone and those treated with CTX plus SAGP (data not shown). At present, there is no direct evidence linking the reduction in the cAMP level to the cell growth-inhibitory action of SAGP. Other effector enzymes in the intracellular transduction system such as phospholipase C and phospholipase $A_2$, which are associated with Gi protein

(Hepburn et al., 1987; Gilman, 1987; Kurachi et al., 1989; Birnbaumer et al., 1990; Bourne et al., 1990; Spriggs et al., 1990; Nakahata et al., 1991), might be linked to the Gi protein modulated by SAGP.

At present, we do not know why the SAGP activity was augmented by CTX in Meth A cells. As Kahn and Gilman (1984) pointed out, ADP ribosylation of Gs promotes the dissociation of its $\alpha$- and $\beta\gamma$-subunits. The $\beta\gamma$-subunits of Gs share identity with those of Gi (Gilman, 1984, 1987) and play practical roles in transmembrane signal cascades (Neer and Clapham, 1988; Birnbaumer et al., 1990; 1992). Therefore, the continuous stimulation of Gs protein by CTX might induce an alteration of IAP-sensitive G-protein or effector molecules linked to the G-protein through its dissociated $\beta\gamma$-subunits. It is possible that there is a mutual regulation between different G-protein-mediated signal pathways. In addition, in the examination of CTX-catalysed ADP ribosylation (Figure 8), two bands of 45 and 47 kDa (Gs$\alpha$) were detected in Meth A cell membrane; these two bands most likely represent the long- and short-splice variants of Gs, whereas only a single band of 47 kDa was present in L929 cell membrane. Furthermore, CTX-catalysed ADP ribosylation seems to increase in the membrane of Meth A cells incubated with IAP (the third lane to the left) as compared with that of control cells, whereas such an effect was not apparent in L929 cell membrane. The difference in responsiveness of Meth A and L929 cells to CTX-catalysed ADP ribosylation may be caused by a difference in their sensitivity to SAGP and the enhanced effect of CTX on the SAGP-induced growth inhibition on Meth A cells. More extensive studies are required to test this hypothesis. Although, our interpretation of the results of the CTX and CTX-catalysed ADP ribosylation assay remains speculative, it is interesting to note that CTX augmented an external signal effect that was inhibited by IAP.

In conclusion, our findings provide the first evidence that an IAP-sensitive GTP-binding protein is involved in the cell growth-inhibitory action of SAGP. SAGP may be useful for the investigation of the signal transduction pathways in cell proliferation, and may be a novel bacterial anti-tumour glycoprotein in cancer therapy because of its immunomodulating and cell growth-inhibitory effects.

### References

AGARWAL S, DRYSDALE B-E AND SHIN HS. (1988). Tumor necrosis factor-mediated cytotoxicity involves ADP-ribosylation. *J. Immunol.*, **140**, 4187–4192.

BIRNBAUMER L. (1992). Receptor-to-effector signaling through G proteins: Roles for $\beta\gamma$ dimers as well as $\alpha$ subunits. *Cell*, **71**, 1069–1072.

BIRNBAUMER L, ABRAMOWITZ J AND BROWN AM. (1990). Receptor-effector coupling by G proteins. *Biochimi. Biophys. Acta.*, **1031**, 163–224.

BOURNE HR, SANDERS DA AND MCCORMICK F. (1990). The GTPase superfamily: a conserved switch for diverse cell functions. *Nature*, **348**, 125–132.

GILMAN AG. (1984). G proteins and dual control of adenylate cyclase. *Cell*, **36**, 577–579.

GILMAN AG. (1987). G proteins: Transducers of receptor-generated signals. *Annu. Rev. Biochem.*, **56**, 615–649.

HEPBURN A, BOEYNAEMS J-M, FIERS W AND DUMONT JE. (1987). Modulation of tumor necrosis factor-$\alpha$ cytotoxicity in L929 cells by bacterial toxins, hydrocortisone and inhibitors of arachidonic acid metabolism. *Biochem. Biophys. Res. Comm.*, **149**, 815–822.

HEPLER JR AND GILMAN AG. (1992). G proteins. *Trends. Biochem. Sci.*, **17**, 383–387.

IMAMURA K, SHERMAN ML, SPRIGGS D AND KUFE D. (1988). Effect of tumor necrosis factor on GTP binding and GTPase activity in HL-60 and L929 cells. *J. Biol. Chem.*, **263**, 10247–10253.

KAHN RA AND GILMAN AG. (1984). ADP-ribosylation of Gs promotes the dissociation of its $\alpha$ and $\beta$ subunits. *J. Biol. Chem.*, **259**, 6235–6240.

KANAOKA M, FUKITA Y, TAYA K, KAWANAKA C, NEGORO T AND AGUI H. (1987*a*). Antitumor activity of streptococcal acid glycoprotein produced by *Streptococcus pyogenes*, Su. *Jpn. J. Cancer Res. (Gann)*, **78**, 1409–1414.

KANAOKA M, KAWANAKA C, NEGOROT T, FUKITA Y AND TAYA K. (1987*b*). Cloning and expression of the antitumor gene of *Streptococcus pyogenes* Su in *Escherichia coli*. *Agric. Biol. Chem.*, **51**, 2641–2648.

KATADA T AND UI M. (1982). Direct modification of the membrane adenylate cyclase system by islet-activating protein due to ADP-ribosylation of a membrane protein. *Proc. Natl Acad. Sci. USA.*, **79**, 3129–3133.

KATADA T, OINUMA M AND UI M. (1986). Two guanine nucleotide-binding proteins in rat brain serving as the specific substrate of islet-activating protein, pertussis toxin. *J. Biol. Chem.*, **261**, 8182–8191.

KURACHI Y, ITO H, SUGIMOTO T, SHIMIZU T, MIKI I AND UI M. (1989). Arachidonic acid metabolites as intracellular modulators of the G protein-gated cardiac $K^+$ channel. *Nature*, **337**, 555–557.

LAEMMLI UK. (1970). Cleavage of structural proteins during the assembly of the head of bacteriophage T4. *Nature*, **227**, 680–685.

MURAYAMA T AND UI M. (1983). Loss of the inhibitory function of the guanine nucleotide regulatory component of adenylate cyclase due to its ADP ribosylation by islet-activating protein, pertussis toxin, in adipocyte membranes. *J. Biol. Chem.*, **258**, 3319–3326.

NAKAHATA N, ABE MT, MATSUOKA I, ONO T AND NAKANISHI H. (1991). Adenosine inhibits histamine-induced phosphinositide hydrolysis mediated via pertussis toxin-sensitive G protein in human astrocytoma cells. *J. Neurochem.*, **57**, 963–969.

NAUTS HC, SWIFT WE AND COLEY BL. (1946). The treatment of malignant tumor by bacterial toxins as developed by the late William B Coley, M.D., reviewed in the light of modern research. *Cancer Res.*, **6**, 205–216.

NEER EJ AND CLAPHAM DE. (1988). Roles of G protein subunits in transmembrane signalling. *Nature*, **333**, 129–134.

NORTHUP JK, STERNWEIS PC, SMIGEL MD, SCHLEIFER LS, ROSS EM AND GILMAN AG. (1980). Purification of the regulatory component of adenylate cyclase. *Proc. Natl Acad. Sci. USA.*, **77**, 6516–6520.

OKAOMOTO H, SHOIN S AND KOSHIMURA S. (1978). Streptolysin s-forming and antitumor activities of group A streptococci. In *Bacterial Toxins and Cell Membranes*, Jeljaszewicz J and Wadström T. (eds) pp. 259–289. Academic Press: London.

OLD LJ. (1985). Tumor necrosis factor (TNF). *Science*, **230**, 630–632.

REILLY HC. (1953). Microbiology and cancer therapy: a review. *Cancer Res.*, **13**, 821–834.

ROOK G. (1992). Tumours and Coley's toxins. *Nature*, **357**, 545.

SPRIGGS DR, SHERMAN ML, IMAMURA K, MOHRI M, RODRIGUEZ C, ROBBINS G AND KUFE DW. (1990). Phospholipase $A_2$ activation and autoinduction of tumor necrosis factor gene expression by tumor necrosis factor. *Cancer Res.*, **50**, 7101–7107.

STARNES CO. (1992). Coley's toxins in perspective. *Nature*, **357**, 11–12.

YOSHIDA J, YOSHIMURA M, TAKAMURA S AND KOBAYASHI S. (1985). Purification and characterisation of an antitumor principle from *Streptococcus hemolyticus* (Su strain). *Jpn. J. Cancer Res.*, **76**, 213–223.

YOSHIDA J, TAKAMURA S AND SUZUKI S. (1987). Cell growth-inhibitory action of SAGP, an antitumor glycoprotein from *Streptococcus pyogenes* (Su strain). *Jpn. J. Pharmacol.*, **45**, 143–147.

YOSHIDA J, TAKAMURA S AND SUZUKI S. (1989). A simplified method for purification of an antitumor acidic glycoprotein from *Streptococcus pyogenes* (Su strain) by immunoadsorbent chromatography. *J. Antibiotics*, **42**, 448–453.

YOSHIDA J, TAKAMURA S AND SUZUKI S. (1991). Antitumor action of an acidic glycoprotein (SAGP) from *Streptococcus pyogenes* in mice. *Biotherapy*, **3**, 331–336.

YOSHIDA J, TAKAMURA S AND SUZUKI S. (1994). Evidence for the involvement of sulfhydryl groups in the expression of antitumor activity of streptococcal acid glycoprotein (SAGP) purified from crude extract of *Streptococcus pyogenes*. *Anticancer Res.*, **14**, 1833–1838.